# Human rotavirus C strain detected in wastewater in Leuven, Belgium

**DOI:** 10.1128/mra.00665-26

**Published:** 2026-07-24

**Authors:** Amal Hosayn, Elke Wollants, Mandy Bloemen, Emmanuel André, Marc Van Ranst, Mustafa Karatas, Jelle Matthijnssens

**Affiliations:** 1 Faculty of Medicine, KU Leuven 26657, Leuven, Belgium; 2 Laboratory of Clinical and Epidemiological Virology, Rega Institute, KU Leuven 26657, Leuven, Belgium; 3 Laboratory of Clinical Microbiology, KU Leuven 26657, Leuven, Belgium; Queens College Department of Biology, Queens, New York, USA

**Keywords:** hybrid-capture, viral metagenomics, environmental surveillance, wastewater sequencing, rotavirus C

## Abstract

Surveillance of urban wastewater in Leuven, Belgium, detected human rotavirus C (RVC) in April 2025. Our analyses revealed a G4P[2] strain closely related to RVC strains detected in feces of schoolchildren in China in March 2025.

## ANNOUNCEMENT

Rotavirus C (RVC) belongs to the *Rotavirus* genus within the family *Sedoreoviridae*. RVC infects several animal hosts, with swine considered the major reservoir (1), whereas human infections are most commonly associated with the G4P[2] genotype (2–4). RVC is believed to mainly circulate in Asia, causing sporadic outbreaks of acute gastroenteritis (1). Evidence of human RVC circulation in Europe remains limited, with only a small number of reported cases in Spain (5), Italy (6), and Hungary (7), all of which occurred before 2014. The RVC genome consists of 11 segments of double-stranded RNA, encoding six structural proteins (VP1–VP4, VP6, and VP7) and five nonstructural proteins (NSP1–NSP5) (8, 9). The outer capsid proteins VP7 and VP4 are used for the classification of RVC into G and P genotypes, respectively (10).

From our recent study (11), we selected the 7 April 2025 wastewater sample with the highest RVC read count for genome construction; RVC was detected in samples collected 1 week before and 1 week after this date as well. The sample was collected from influent wastewater at Aquafin Leuven, Belgium (50.902098°N, 4.711509°E), using 24-h composite sampling. Briefly, 500 mL of wastewater was concentrated by 30 kDa ultrafiltration, then RNA was extracted from 350 µL of concentrate using the RNeasy Mini Kit (Qiagen, Germany), cDNA was constructed with ProtoScript II (New England Biolabs, USA), and libraries were prepared with the Twist Library Preparation EF 2.0 Kit (Twist Bioscience, USA), enriched with the Twist Comprehensive Viral Panel (Twist Bioscience), and sequenced on the AVITI platform (Element Biosciences; 2 × 150 bp). Reads were processed with fastp (v0.23.2) for deduplication and quality control with EsViritu (v0.2.3) (12). A total of 47.6 million reads were used for downstream analysis. Reference-guided assembly was performed in Geneious Prime v2025.2.2 using Geneious Mapper with the Medium-Low Sensitivity/Fast setting and reference sequences obtained from NCBI GenBank. The assembled sequences for all 11 segments were compared with other RVC sequences using NCBI BLASTN.

**Fig 1:**
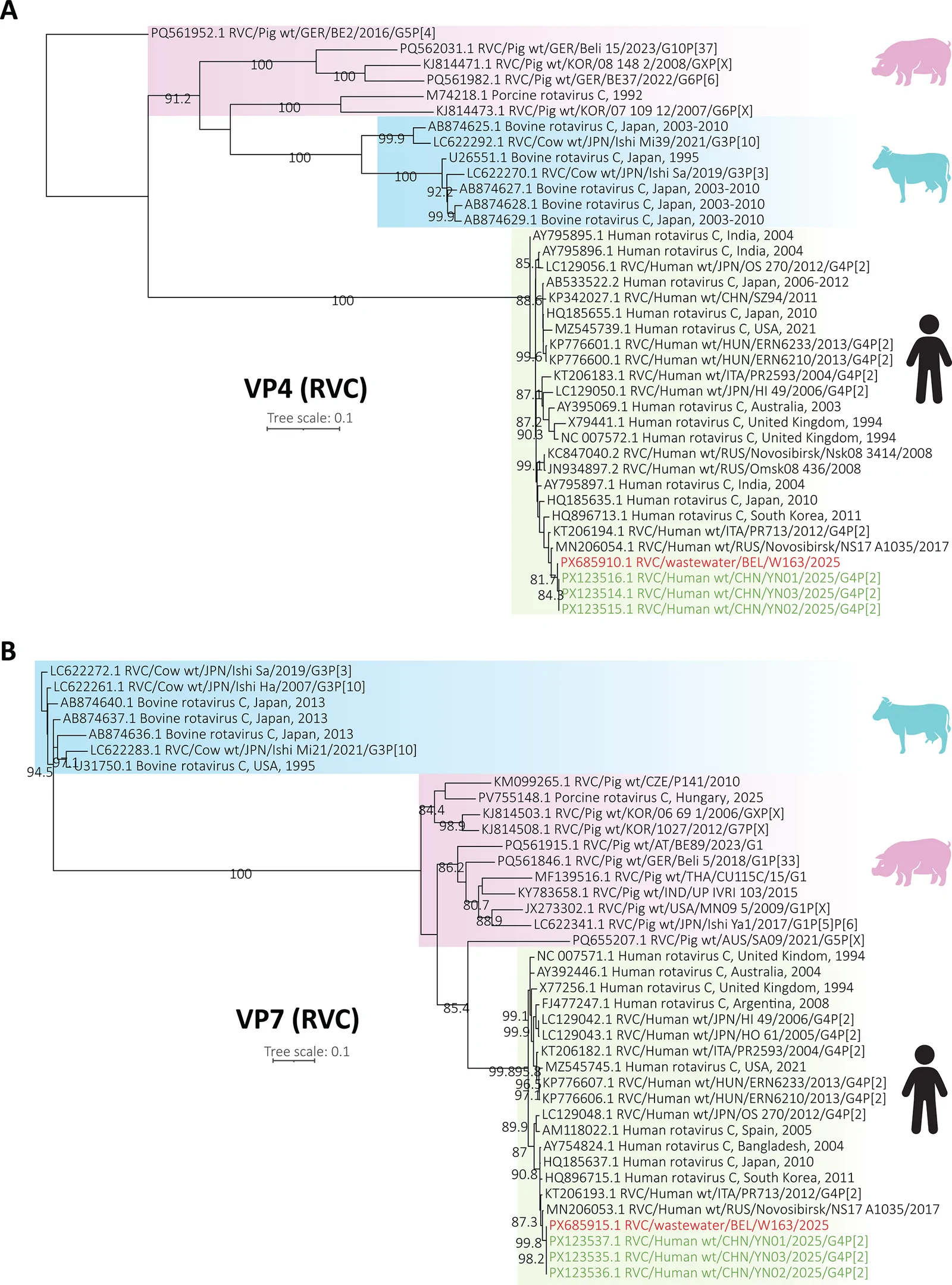
Phylogenetic analysis of VP4 (**A**) and VP7 (**B**) genes at the nucleotide level. The assembled sequences were BLASTed against GenBank, after which multiple RVC reference sequences for each segment were downloaded (November 2025). BLAST hits were first sorted by percent identity. The 10 best hits were selected after reducing redundancy among identical sequences, and additional non-human RVC sequences were then randomly selected from the remaining list to provide representative diversity while removing redundant sequences. The downloaded sequences were subsequently aligned together with the assembled sequences from Leuven (highlighted in red) using the MAFFT tool in Geneious Prime. A maximum-likelihood phylogenetic analysis, based on nucleotide alignments of the VP4 and VP7 genes from 38 and 39 rotaviruses, respectively, was performed in Geneious Prime using PhyML with the HKY85 substitution model and 1,000 bootstrap iterations. The assembled VP4 sequence (indicated in red in panel **A**) and VP7 sequence (indicated in red in panel **B**) clustered within the human RVC genotype G4P[2], closely related to several RVC strains from China (indicated in green).

We detected a human RVC strain in Leuven wastewater, representing a catchment area of >100,000 individuals. BLASTN analysis of each of the 11 assembled sequences revealed high nucleotide similarity (98.1%–100%) to several RVC strains from China isolated in March 2025, including RVC/Human-wt/CHN/YN03/2025/G4P[2], which were detected in feces of schoolchildren, with genotype G4P[2]-I2-R2-C2-M2-A2-N2-T2-E2-H2 (Table 1). Moreover, all 11 gene segments of the RVC strain detected in Leuven wastewater clustered with human RVC strains, further confirming that it is a human-infecting strain with genotype G4P[2]. VP7 and VP4 clustering patterns are shown in Fig. 1A and B. This detection in Belgian wastewater occurred around the same time as detection of related RVC strains in China, where GenBank records describe an outbreak of gastroenteritis among school-aged children (Table 1). On the whole, we want to bring attention to the viruses that are potentially circulating under the radar while causing detectable outbreaks in some regions around the world.

**TABLE 1:** Genomic characteristics and sequence similarity of the Leuven wastewater RVC strain compared with closest complete GenBank sequences^*a*^

Segment no.	Genotype	Protein	CDS length (bp)	GC content (%)	Sequencing coverage depth on average (×)	Completeness (%)	GenBank accession number	Closest relative sequence in GenBank	Similarity to the GenBank sequence (%)
1	R2	VP1	3,191	30.4	121.1	96.4	PX685907	PX123506.1	99.8
2	C2	VP2	2,655	31.25	68.7	100	PX685908	PX123508.1	99.7
3	M2	VP3	2,082	29.2	87.2	100	PX685909	PX123511.1	99.7
4	P(2)	VP4	2,026	31.15	189.1	88.7	PX685910	PV870548.1	99.8
5	A2	NSP1	1,185	29.03	30.4	100	PX685911	PX123520.1	100.00
6	I2	VP6	1,090	33.26	83.1	80.6	PX685912	PX123517.1	99.1
7	T2	NSP3	1,215	32.02	531.6	100	PX685913	PX123526.1	99.7
8	N2	NSP2	954	32.49	290.4	100	PX685914	PX123523.1	100
9	G4	VP7	986	32.35	66.3	92.8	PX685915	PV870554.1	100
10	E2	NSP4	416	29.57	179	67.9	PX685916	PX123530.1	100
11	H2	NSP5	626	32.59	81.5	85.7	PX685917	PX123532.1	99.7

^*a*^ Segment completeness was determined by calculating the ratio between the assembled segment length and the corresponding reference genome length, then multiplying it by 100. The GC content was calculated using the “GC content calculator” on the website VectorBuilder (https://en.vectorbuilder.com/).

## Data Availability

The (partial) coding sequence data for RVC isolate RVC/wastewater/BEL/W163/2025 have been deposited in the NCBI GenBank database under accession numbers PX685907, PX685908, PX685909, PX685910, PX685911, PX685912, PX685913, PX685914, PX685915, PX685916, and PX685917. Raw data are available at SRA (Sequence Read Archive) under accession number SRS28263951. Reference-guided assembly was conducted using the sequences available on GenBank (accession numbers: PX123505.1, NC_007546.1, NC_007574.1, PX123514.1, NC_007544.1, PX123517.1, NC_007543.1, NC_007545.1, NC_007571.1, PX123530.1, and PX123532.1).
